# Associations between Housing Insecurity and Physiological Health among Aging Adults in the United States

**DOI:** 10.1093/geroni/igaf122.392

**Published:** 2025-12-31

**Authors:** Aarti Bhat, Andrew Fenelon, Jessica Ho, Idan Shalev, David Almeida

**Affiliations:** The Pennsylvania State University, University Park, Pennsylvania, United States; University of Minnesota, Minneapolis, Minnesota, United States; The Pennsylvania State University, University Park, Pennsylvania, United States; The Pennsylvania State University, University Park, Pennsylvania, United States

## Abstract

Housing insecurity (HI) is a stressor that may have implications for physiological health. While extant literature has examined relationships between HI and physiological markers, these studies are limited by their cross-sectional nature and examine only composite allostatic load scores or individual physiological markers as outcomes of interest. In contrast, this study utilizes longitudinal data from the Midlife in the United States (MIDUS) study to assess associations between HI and composite allostatic load scores, as well as individual physiological subsystem scores that comprise allostatic load; and utilizes allostatic load scores from the prior wave of MIDUS to control for health selection. The analytic sample comprised 448 adults (mean age = 64.94, 55.80% female, 15.85% Black, 6.70% other non-white race). Controlling for sociodemographic and health covariates as well as prior wave inflammatory subsystem scores, HI was associated with significantly higher levels of inflammation; however, HI was not associated with overall allostatic load nor other physiological subsystems. We examined whether perceived and objective neighborhood quality moderated the association between HI and inflammation. With increased levels of HI, lower perceived neighborhood quality and higher scores of objective neighborhood deprivation were significantly associated with higher inflammation. Among participants who reported experiencing at least one HI event, those who rented their homes experienced higher inflammation compared to those who owned their homes outright. These analyses indicate inflammation may be a main biological pathway impacted by HI in aging adults, and have implications for health-related interventions to mitigate adverse health effects of HI in this vulnerable population.

